# Biting behaviour of African malaria vectors: 1. where do the main vector species bite on the human body?

**DOI:** 10.1186/s13071-015-0677-9

**Published:** 2015-02-04

**Authors:** Leo Braack, Richard Hunt, Lizette L Koekemoer, Anton Gericke, Givemore Munhenga, Andrew D Haddow, Piet Becker, Michael Okia, Isaac Kimera, Maureen Coetzee

**Affiliations:** Centre for Sustainable Malaria Control, University of Pretoria, Pretoria, South Africa; Zoonoses Research Unit, University of Pretoria, Pretoria, South Africa; Wits Research Institute for Malaria, Faculty of Health Sciences, University of the Witwatersrand, Johannesburg, South Africa; Vector Control Reference Unit, Centre for Opportunistic, Tropical and Hospital Infections, National Institute for Communicable Diseases, National Health Laboratory Service, Johannesburg, South Africa; Avima (Pty) Ltd, 18 Aschenberg Street, Chamdor, Gauteng South Africa; Centre for Biodefence and Emerging Infectious Diseases, The University of Texas Medical Branch, Galveston, TX USA; Centre for Sustainable Malaria Control, University of Pretoria, Pretoria, South Africa; National Malaria Control Programme, Kampala, Uganda

**Keywords:** *Anopheles*, Biting behaviour, Feeding behaviour, Outdoor biting, Malaria

## Abstract

**Background:**

Malaria control in Africa relies heavily on indoor vector management, primarily indoor residual spraying and insecticide treated bed nets. Little is known about outdoor biting behaviour or even the dynamics of indoor biting and infection risk of sleeping household occupants. In this paper we explore the preferred biting sites on the human body and some of the ramifications regarding infection risk and exposure management.

**Methods:**

We undertook whole-night human landing catches of *Anopheles arabiensis* in South Africa and *Anopheles gambiae s.s.* and *Anopheles funestus* in Uganda, for seated persons wearing short sleeve shirts, short pants, and bare legs, ankles and feet. Catches were kept separate for different body regions and capture sessions. All *An. gambiae s.l.* and *An. funestus* group individuals were identified to species level by PCR.

**Results:**

Three of the main vectors of malaria in Africa (*An. arabiensis, An. gambiae s.s.* and *An. funestus*) all have a preference for feeding close to ground level, which is manifested as a strong propensity (77.3% – 100%) for biting on lower leg, ankles and feet of people seated either indoors or outdoors, but somewhat randomly along the lower edge of the body in contact with the surface when lying down. If the lower extremities of the legs (below mid-calf level) of seated people are protected and therefore exclude access to this body region, vector mosquitoes do not move higher up the body to feed at alternate body sites, instead resulting in a high (58.5% - 68.8%) reduction in biting intensity by these three species.

**Conclusions:**

Protecting the lower limbs of people outdoors at night can achieve a major reduction in biting intensity by malaria vector mosquitoes. Persons sleeping at floor level bear a disproportionate risk of being bitten at night because this is the preferred height for feeding by the primary vector species. Therefore it is critical to protect children sleeping at floor level (bednets; repellent-impregnated blankets or sheets, etc.). Additionally, the opportunity exists for the development of inexpensive repellent-impregnated anklets and/or sandals to discourage vectors feeding on the lower legs under outdoor conditions at night.

## Background

The renewed commitments to global malaria eradication [1-3] and vigorous interventions towards that goal over the past decade have resulted in a dramatic decline in case numbers and mortalities associated with this disease [4-6]. However, challenges, especially in Africa, associated with increasing vector resistance to current-generation insecticides [5,7-9], shifting vector behaviour towards increased outdoor biting thus avoiding insecticide-treated surfaces [10-14], and shortfalls in funding [5] collectively mean that a plateau in the decline of malaria may be reached, or at least a reduction in the pace of malaria control success. The current near-complete reliance on indoor residual spraying (IRS) and insecticide treated bed nets (ITN’s) for vector control is a cause for concern [5] and lends support for calls advocating broadened control strategies and exploration of fresh approaches [15-17]. In this paper we focus attention on the unusually similar biting behaviour of three of the most important African malaria vectors, *Anopheles gambiae s.s*., *An. arabiensis* and *An. funestus*. We demonstrate the strong preference for feeding on the human body at very low levels close to the ground, and discuss the opportunities this presents for behavioural targeting to reduce biting risk and therefore reduced malaria infection.

## Methods

### Study areas

For *An. arabiensis* we selected Malahlapanga (S22°53.374′ E31°02.391′) in South Africa as this site usually has a strong, reliable population of this species present. It is a freshwater spring in a remote wilderness setting, the site attributes described by Braack *et al.* [18]. Because there are no human dwellings in the vicinity, only outdoor catches were possible.

For *An. gambiae* and *An. funestus,* we worked in a high-transmission malaria region in northern Uganda, based on the advice of the Uganda National Malaria Control Programme staff. Most collections were made in or very near the villages of Agule (N01°41.130′ E33°12.944′), Akaidebe (N02°06.953′ E33°00.372′) and Araki (N02°10.879′ E32°55.979′).

### Mosquito collectors

In South Africa, we used persons well experienced in human landing catches, drawn from malaria research institutions and Provincial Malaria Control Programmes. In Uganda we relied on a mix of well-experienced malaria entomology researchers and Uganda National Malaria Control staff supplemented occasionally with local villagers trained to do human landing catches. For such newly trained villagers, the first few nights of catches were disregarded until they were considered sufficiently experienced. Senior research staff were present at all times either as part of the group doing collections (small group focal collections) or walking between different groups for whole-night quality control of collectors placed inside and outside dwellings in villages.

### Study design

Our primary focus was to understand where on the human body the main vector species bite, especially in outdoor situations. We therefore placed trained mosquito catchers—in some situations well away from any village or human dwellings or in some cases within a village but at least three metres from the nearest human dwelling—seated on plastic stools or chairs and spaced at least three metres apart. At first, we wore only short pants (i.e. no shirt or socks and shoes), but as it became apparent after several hundred bites that only the lower legs were being targeted by vector species we reverted to wearing short-sleeved shirts, short pants with bare legs and feet; this also enabled us to bring women volunteers into the study as catchers as any concerns about naked torsos were then obviated. All people were requested not to apply repellents or deodorants or other odour substances within at least 12 hours of commencing catches. All persons were trained in human landing catches (HLC) and represented a mix of males and females, black and Caucasian, between 18 to 60 years of age. Each person was provided with a flashlight, an aspirator to catch biting mosquitoes, and three netting-topped polystyrene cups for each 45-minute catch-session. Each polystyrene mosquito holding cup was labelled with the name of the person, time-session, and body-region at which the mosquito was caught. The body-regions were defined as described below.

For *An. arabiensis* in South Africa where we started this study, polystyrene cups were labelled at first into multiple body regions (see Table 1), but as it involved many cups for multiple persons for multiple catch sessions per night, and because it became apparent that biting was limited to particular body regions, we soon simplified collection cups to reflect only “Ankles/Feet”, “Lower Leg”, and “Rest of Body”. The upper limit of the ankle was arbitrarily defined as being at the narrowest part of the lower leg where the ankle then starts to form a bulge. “Lower Leg” was defined as being from that narrowest part of the leg at the ankle, up to a point halfway to the knee. A line was drawn with a pen on each person’s leg to minimize confusion in interpretation where “Lower Leg” ends.

**Table 1:** **Detailed bite sites for** ***Anopheles arabiensis*** **at people wearing short pants only**

**Toes**	**Foot**	**Ankle**	**Ankle to mid-calf**	**Mid-calf to knee**	**Upper leg**	**Torso**	**Arms & hands**	**Shoulders & neck**	**Head**
9 (8.2%)	27 (24.5%)	59 (53.6%)	13 (11.8%)	2 (1.8%)	0 (0.0%)	0 (0.0%)	0 (0.0%)	0 (0.0%)	0 (0.0%)

Number (and percentage) of bites on bodies of three seated persons during one night; no shirt, short pants only, no socks or shoes (n = 110 bites).

For *An. gambiae s.s*. and *An. funestus* in Uganda, initially we followed the same categorization of catches as for *An. arabiensis* in South Africa, but later when the feeding trends became apparent we further simplified the catch-categories into “Lower Legs” (which included ankles and feet) and “Rest of Body”. When villagers were assisting with catches, we drew pictures of Lower Leg and Rest of Body on the cups for easier identification of cups.

Catch data for *An. arabiensis* include results from intensive work at Malahlapanga from 1990 to 1992 but with supporting catches during November and December 2012. Data for *An. gambiae* s.s. and *An. funestus* are from two months of work in Uganda during April and October 2013.

Mosquito collections took place from 18 h00 to 06 h00, except on two nights when only a few hours were possible due to onset of rain and/or wind (Ubuli and Ogobi, Table 2). Catch-sessions lasted for 45-minute periods every hour, with a 15 minute break to allow changes in collection cups, refreshments and ablutions, before commencing the next 45-minute session. Each mosquito was blown from the aspirator into the appropriate cup labelled as to body region. All mosquitoes were kept in cool-boxes to maximise survival, then killed upon arrival back at the central workstation by freezing, microscopically separated into species (*An. gambiae* complex or *An. funestus* group), counts recorded on paper-forms for later transfer to computer spreadsheet, and each mosquito of *gambiae* complex or *funestus* group individually placed in an Eppendorf tube with silica gel. Preserved mosquitoes were subsequently identified by PCR assays at the laboratories of the Wits Research Institute for Malaria, University of the Witwatersrand, Johannesburg, South Africa. Of the mosquitoes historically known as *An. gambiae s.s*. “M” or “S” molecular forms, only one individual was identified as “M” form (from Uganda), now named *An. coluzzii* Coetzee & Wilkerson [19]. All references to *An. gambiae s.s*. in this paper relate only to the “S” molecular form (now known as the nominotypical *An. gambiae* Giles).

**Table 2:** **Distribution of bites by** ***Anopheles gambiae*** **s.s. and** ***Anopheles funestus***

	***Anopheles gambiae s.s.***			***Anopheles funestus***		
**Locality**	**Ankle/foot**	**Ankle to mid-calf**	**Rest of body**	**Ankle/foot**	**Ankle to mid-calf**	**Rest of body**
Araki night 1	9	4	0	15	1	0
Araki night 2	15	3	0	14	4	0
Araki night 3	7	2	0	18	2	0
Araki night 4	4	0	0	2	0	0
Araki night 5	7	1	0	7	1	0
Araki night 6	5	3	0	5	0	0
Ubuli	1	0	0	1	0	0
Ogobi	2	0	0	0	0	0
Agule	14	1	0	14	0	0
**Total**	**64**	**14**	**0**	**76**	**8**	**0**

Two persons seated outdoors away from village, wearing short-sleeve shirt, short pants, no socks or shoes. North-central Uganda.

For each of the three vector species discussed in this paper, at some point it became clear that there was a definite preference for feeding at ankles and feet of standing or seated people. To test the effect of denying access by vector mosquitoes to such high-preference ankles and feet regions, we covered this area with plastic bags for complete exclusion. This was done both in South Africa and in Uganda when a sufficient number of people were available, usually by having at least one person with ankles and feet covered in the group, and different people taking turns on different nights to randomize attractiveness of different people. Such persons with covered ankles and feet would sit the whole night following the same routine of mosquito landing catches as persons with exposed ankles and feet. The purpose of the exercise was to determine the effect on biting-rate and potential bite-site shifts arising from preventing *Anopheles* accessing the preferred ankles and feet positions (i.e. do those mosquitoes that would normally bite on ankles and feet move to other parts of the body if denied access to ankles and feet).

To understand biting patterns on sleeping people, we placed individual persons wearing only short pants (no foot covering) on mats on the ground, with two or more collectors roaming around the body of the reclining person to catch landing mosquitoes. Such mosquitoes were kept in polystyrene containers and treated in the same way as outlined above for seated people. The roaming collectors stood well away (c.a. three metres) from the persons lying on the ground so as to avoid their lower legs attracting or otherwise affecting mosquitoes at the recumbent persons, moving in to collect mosquitoes only when prompted by the recumbent persons that they felt a mosquito biting them.

In summary, we had six categories of human landing catches:

- Seated people wearing short pants but no shirt and no socks or shoes, far removed from any human habitation;
- Seated people wearing short pants, shirt but no socks and shoes, far removed from any human habitation;
- People seated outdoors within a typical small African village but at least three metres from the nearest dwelling, wearing short pants, shirt but no socks and shoes;
- People seated indoors within a typical small African, wearing short pants, shirt but no socks and shoes;
- Mixed group with all people wearing short pants and shirts, but some having no socks and shoes and some having ankles and feet covered with plastic bags;
- Two people lying outdoors flat on the ground wearing short pants only.

### Data analysis

In this mainly descriptive study use was made of frequencies, proportions, percentages and 95% confidence intervals to describe the data. The comparison of indoor and outdoor biting patterns employed a two-sample proportions test at the 0,05 level of significance.

### Ethical considerations

Ethical clearance was obtained from the University of Pretoria for human landing catches and all other aspects of this study, thereby also satisfying the ethical clearance requirements of collaborating institutions in South Africa and Uganda. In Uganda we worked in collaboration with staff of the Uganda National Malaria Control Programme, through whom permission was obtained from village leaders and household heads to work in specific areas and to obtain volunteers for training as mosquito collectors. Informed consent signatures were obtained from villagers who assisted with the human landing catches. All persons conducting mosquito human landing catches were provided with free malaria prophylactic medication.

## Results

### South Africa: *Anopheles arabiensis*

#### Human body feeding site preferences

Of 110 *An. arabiensis* caught outdoors feeding on seated humans wearing only short pants but no shirt, socks or shoes, 86.4% (95/110; 95% CI = 80.0% - 92.8%) landed and commenced feeding at toes, feet and ankles, while the remaining bites were above the ankles but below the knee (see Table 1).

Of 1,614 *An. arabiensis* caught outdoors feeding on seated humans wearing T-shirts, short pants but no socks or shoes, 92.7% (1496/1614; 95% CI = 91.4% - 94.0%) landed and commenced feeding at toes, feet and ankles, while another 5.0% attempted feeding immediately above the ankle below mid-calf level. Thus, 97.7% (1577/1614; 95% CI = 97.0% - 98.4%) of *An. arabiensis* were biting at the very lowest part of the body (the remaining 2.3% fed below the knees) (see Table 3).

**Table 3:** **Detailed bite sites for** ***Anopheles arabiensis*** **at people wearing shorts and shirts**

**Toes**	**Foot**	**Ankle**	**Ankle to mid-calf**	**Mid-calf to knee**	**Upper leg**	**Torso**	**Arms & hands**	**Shoulders & neck**	**Head**
203 (12.6%)	484 (29.9%)	809 (50.1%)	81 (5.0%)	37 (2.3%)	0 (0.0%)	0 (0.0%)	0 (0.0%)	0 (0.0%)	0 (0.0%)

Number (and percentage) of bites on bodies of four seated persons over six nights, wearing T-shirt, short pants, no socks or shoes (n = 1 614 bites).

Based on a larger sample (not necessarily focussed on bite site preference but other studies to determine nocturnal biting cycle) of 2,181 *An. arabiensis* caught at monthly sampling sessions during the hot season (October to April) and straddling the wet season (November to March), the nightly human biting rate was 25.96 which is the average number of bites a person could expect to receive per night from *An. arabiensis* at Malahlapanga during that season.

**Figure 1:**
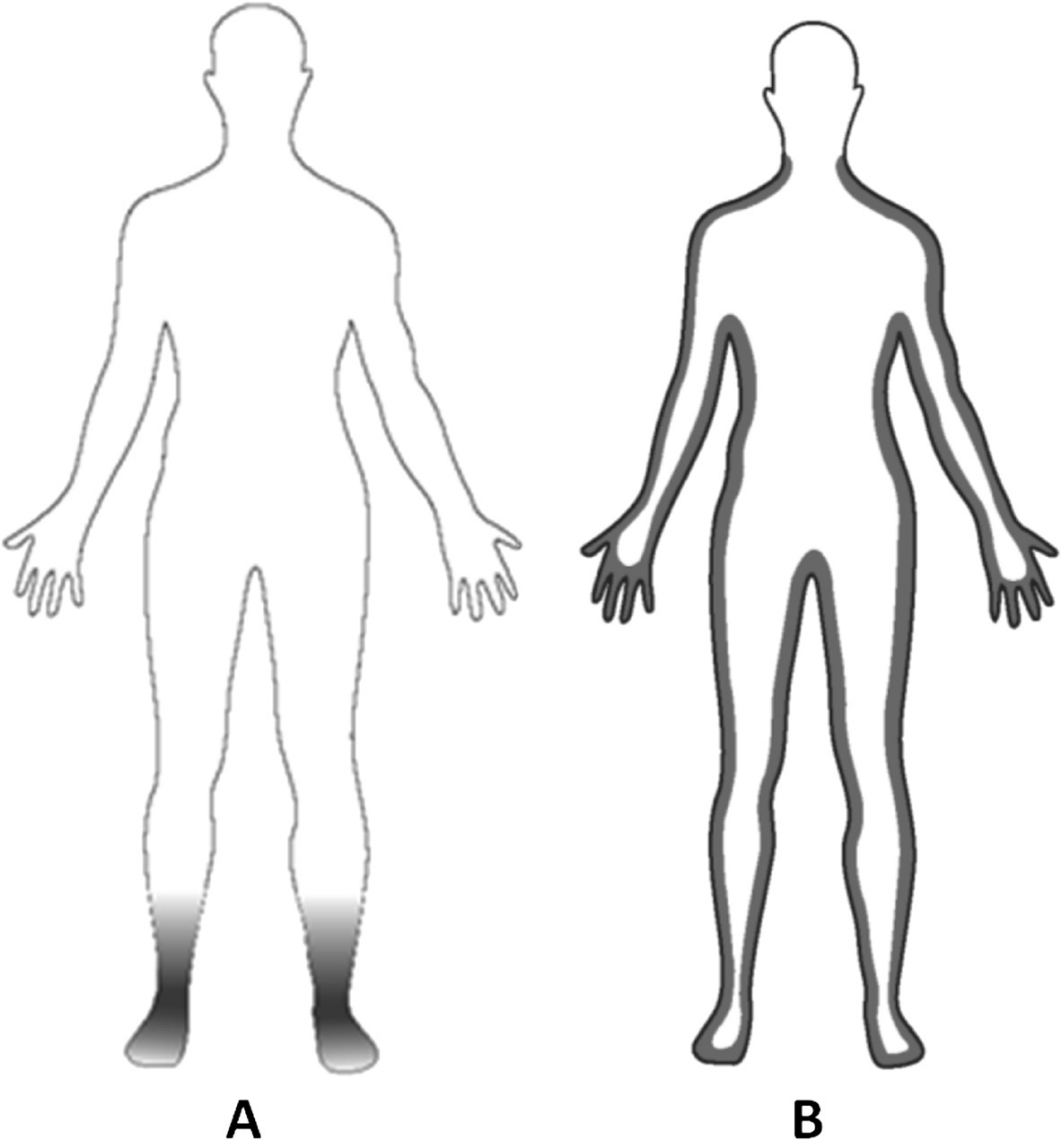
**Preferred bite sites of** ***Anopheles arabiensis*** **,** ***An. gambaie*** **and** ***An. funestus*** **on the human body.** Darkened areas represent the preferred areas of all three species for biting on the human body, at **(A)** standing or seated humans and **(B)** at people lying flat on the ground.

To better understand whether the strong preference for feeding at ankles and feet related to an intrinsic attraction to these body regions or more indirectly related to height above ground, we placed people lying flat on the ground, wearing only short pants. Under such conditions *An. arabiensis* landed and commenced feeding within a few centimetres of almost all points of contact between the body and ground, excluding the head (n = 158 bites). Distribution of bites is provided in Table 4 and graphically represented in Figure 1.

**Table 4:** **Number of bites at body regions of persons lying flat on ground**

**Species**	**Ankles & feet**	**Above ankles to knee**	**Upper leg**	**Torso**	**Arms and hands**	**Shoulders and neck**	**Head**
*An. arabiensis*	41	24	28	26	32	7	0
	**Lower leg**	**Rest of body**					
*An. gambiae*	22	49					
*An. funestus*	12	41					

Bites recorded from two people on one night; *An. arabiensis* in South Africa, *An. gambiae* and *An funestus* in Uganda.

### Effect of denying access to ankles and feet

Using ten people as bait over a series of nights, five persons with covered ankles/feet alternating with five people having exposed ankles/feet, a total of 1,118 bites were received from *An. arabiensis*. Of these, 852 were at persons with exposed ankles/feet and 266 at persons with covered ankles/feet (bites immediately above plastic bag). This translates to a 68.8% (586/852; 95% CI = 65.7% - 71.9%) reduction in the number of bites by *An. arabiensis* at people having protected ankles and feet, under the assumption that if uncovered both groups will have received the same number of bites, i.e. n = 852.

### Uganda: *Anopheles gambiae* and *Anopheles funestus*:

The findings for *An. gambiae* and *An. funestus* were in principle the same as for *An. arabiensis*.

At people seated in an open clearing some 200 m from a small village, 82.1% (64/78; 95% CI = 73.5% - 90.6%) of *An. gambiae* landed and commenced feeding at ankles and feet, compared with 90.5% (76/84; 90% CI = 84.2% - 96.8%) of *An. funestus* landing and feeding at ankles and feet. For both species, 100% of bites occurred below mid-calf level (Table 2). At people seated outdoors in a small village at least three metres from the nearest hut, 95.0% (114/120; 95% CI = 91.1% - 98.9%) of *An. gambiae* (n = 120) and 92.7% (165/178; 95% CI = 88.9% - 96.5%) of *An. funestus* (n = 178) landed and commenced feeding below mid-calf height (all other bites from *An. gambiae* and *An. funestus* at seated people occurred between the knee and mid-calf). The percentages dropped at people seated indoors presumably because of the clutter of furniture and proximity of walls which forced mosquitoes to “bounce” up to overcome obstacles; here 81.4% (201/247; 95% CI = 76.5% - 86.2%) of *An. gambiae* (n = 247) and 77.3% (355/459; 95% CI = 73.5% - 81.2%) of *An. funestus* (n = 459) landed and fed below mid-calf height (remainder below the knees) (Tables 5 and 6). For both species, the proportion of bites on lower legs differed significantly between people seated outdoors and those seated indoors (*An. gambiae* p < 0.001, 95% vs 81.4%; *An. funestus* p < 0.001, 92.7% vs 77.3%). Covering the lower legs (below mid-calf level) of people seated outdoors reduced the number of bites from *An. gambiae* by 58.5%; (27/65; 95% CI = 46.5% - 70.4%) and *An. funestus* by 77.8% (42/54; 95% CI = 66.7% - 88.9%).

**Table 5:** ***Anopheles gambiae s.s.:*** **distribution of bites on people seated outdoors and indoors**

	**Outdoor**		**Indoor**	
**Locality**	**Lower legs (Mid-calf to toes)**	**Rest of body**	**Lower legs (Mid-calf to toes)**	**Rest of body**
Akaidebe	7	0	15	5
Araki Night 1	18	0	49	12
Araki Night 2	32	0	21	6
Araki Night 3	42	5	61	15
Araki Night 4	15	1	55	8
**TOTAL**	**114**	**6**	**201**	**46**

Four persons seated outdoors and four persons indoors within village, wearing short-sleeve shirt, short pants, no socks or shoes. North-central Uganda.

**Table 6:** ***Anopheles funestus:*** **distribution of bites on people seated outdoors and indoors**

	**Outdoor**		**Indoor**	
**Locality**	**Lower legs (Mid-calf to toes)**	**Rest of body**	**Lower legs (Mid-calf to toes)**	**Rest of body**
Akaidebe	6	0	33	12
Araki Night 1	32	1	96	45
Araki Night 2	35	0	41	7
Araki Night 3	63	9	98	23
Araki Night 4	29	3	87	17
**TOTAL**	**165**	**13**	**355**	**104**

Four persons seated outdoors and four persons indoors within village, wearing short-sleeve shirt, short pants, no socks or shoes. North-central Uganda.

Sample size for mosquitoes biting at persons lying flat on the ground wearing only short pants was small (*An. gambiae* n = 71 and *An. funestus* n = 53), but again followed the same trend as for *An. arabiensis*, by not focussing bites strongly at lower legs but biting close to the ground at most parts of the body except the head (Table 4).

### Indoor/Outdoor biting preferences

When comparing Indoor vs Outdoor feeding preference for the two species at village settings in northern Uganda, based on groups of five people seated outdoors and five indoors doing whole-night HLC’s, 32.7% of *An. gambiae* bites occurred outdoors and 67.3% occurred indoors (n = 367). For *An. funestus*, 27.9% of bites occurred outdoors and 72.1% indoors (n = 637) (Tables 5 and 6).

## Discussion

A considerable body of published work exists providing evidence that not only do different mosquito species often prefer to feed at specific body regions [20-24] (Table 7), but also for targeting hosts at preferred heights above ground [18,24-27]. This study shows that there is a remarkable convergence in biting behaviour in at least three of the most important malaria vectors in Africa.

**Table 7:** **Preferred bite sites for various mosquito species (field conditions)**

**Species**	**Preferred bite site**	**Reference**
*Aedes aegypti*	Head & shoulders	20
*Aedes simpsoni*	Head & shoulders	21
*Culex pipiens fatigans*	82% of bites below knee (seated people) 34% of bites below knee (lying down)	22
*Culex quinquefasciatus*	Non-specific whole body	20
*Eretmapodites chrysogaster*	Between ankles & knee	23
*Anopheles atroparvus*	Head & shoulders	20
*Anopheles albimanus*	Head & neck	20
*Anopheles farauti*	Feet	24

### Human body feeding site preferences

The three species *An. gambiae, An. arabiensis* and *An. funestus* investigated in this study all displayed a strong preference for feeding at the lower leg areas at seated people, such sitting simulating typical outdoor social situations in the evening in rural villages in Africa. There was an absolute preference for feeding below the knees, with not a single bite above that height in any of the three species of *Anopheles*, at seated people. The overwhelming majority of bites occurred below mid-calf level, where 97.7% of *An. arabiensis* bites occurred (92.7% specifically at ankles and feet), *An. gambiae* varied between 81.4% - 100% and *An. funestus* 77.3% - 100% preference for below mid-calf biting.

Our findings for *An. arabiensis* are supported by those of Govere *et al.* [28] working with eight human bait subjects (only short pants, no shirt, socks or shoes) at the same Malahlapanga site in South Africa, who found that 97.5% of bites (n = 519) by this species occurred below the knee (81.1% on ankles and feet).

The evidence from this present study also suggests that the preference for feeding especially at the ankle region of seated people is not necessarily targeted at ankles or feet *per se*, but is related to height above ground. While well over 70.0% of bites by all three principal malaria vector species occurred below mid-calf level at people seated on chairs (and 100% below knee level), as soon as people lie down flat on the ground the biting pattern changed, with bites occurring almost anywhere on the body (except the head) near its point of contact with the ground. Dekker *et al.* [29] conducted trials under strict laboratory conditions and found the same trends, whereby seated people received most bites on legs and feet, but at people lying down with raised legs the bites from *An. arabiensis, An. gambiae* and *An. quadriannulatus* (the latter a non-vector species) shifted to the body parts close to the ground while the legs and feet received significantly less bites. These authors concluded that the driving cause for such preference in biting pattern was due to convection currents partially mediated by host odours. We have reservations regarding these conclusions, based on earlier published evidence [18] that clearly showed a rapid drop-off in biting rate by *An. arabiensis* with increasing height above ground, unrelated to potential convection current effects. Similar behaviour has been found in other locations and species, which also demonstrate a preference for feeding close to the ground [24,25,30]. We suggest that the malaria vector species have innate behaviour which drives them to feed preferentially close to ground level, using odours as an initial means to detect and locate potential hosts, but that final selection of bite site is determined by a more complex interaction of cues (including odours, heat, moisture) with height above ground as a primary over-riding factor.

Takken & Knols [31] reviewed the literature regarding odour-mediated behaviour of African malaria vectors and concluded that there is strong evidence that *An. gambiae, An. arabiensis* and *An. funestus* are attracted to human volatiles from a distance. Multiple volatiles have attractant qualities for mosquitoes, some with strong effect on malaria vector species and others not, including that animal (non-human) odours (comprising multiple kairomones) are not particularly attractive to *An. gambiae* while human odours are. The review provides ample evidence that at least initial attraction and orientation for a range of mosquitoes towards a host as a broad target is largely based on odours, and that the feet are often a rich source of some particular volatiles, but it does not go as far as to show what may cause different mosquitoes to preferentially feed at often different body regions. Other studies [31,32] have shown that Limburger cheese is attractive to *An. gambiae* and that the coryneform bacteria responsible for production of the attractant volatiles in such cheese are close relatives of *Brevibacterium epidermidis* which is commonly associated with human feet and produce odours attractive to such mosquitoes. However, our understanding of the role of odour attractants is still incomplete, and although such odours may serve at least as initial cues to bring vectors towards a suitable host for feeding, little is known regarding what causes a mosquito to often zero in on a particular body region, or to feed at an apparent preferred height.

In summary, our work under rural African field conditions confirm the laboratory findings of earlier studies on biting preferences of the main malaria vector species, showing that under typical outdoor socializing situations with people seated on stools and chairs, *An. arabiensis*, *An. gambiae* and *An. funestus* all show a very pronounced clear preference for feeding at the lower-most parts of the body close to the ground. This behaviour changes when people lie down, in which situation biting becomes more random along a band of the body (excluding head) in contact with the ground or resting substrate. While our studies were confined to South Africa and Uganda, conversations with fellow malaria entomologists working elsewhere in Africa suggest that the same biting patterns exist in multiple countries across the continent, and that this is therefore likely to be general, innate behaviour within at least the three species discussed in this paper; clearly, however, this generalization needs to be proved by way of further field studies.

### Effect of denying access to ankles and feet

Another finding of this study is that if the three vector species *An. arabiensis, An. gambiae* and *An. funestus* are denied access to the lowermost parts of the body when people are seated outdoors, that percentage of mosquitoes that would have bitten at feet, ankles and below-calf level do not shift to feed higher up the body, but appear to move away to presumably find another host who does have the preferred feeding stratum exposed. This again reinforces the impression that biting and feeding is strongly correlated with height above ground; why move away if perfectly adequate feeding sites are available a short distance higher? As has already been discussed, these same mosquitoes will readily bite and feed at other sites of the body provided that the host is lying down on the ground. Moreover, raising the body to higher levels above ground results in a sharp decline in bites even at ankles and feet [18]. While this research area appears to have received little attention, at least one other study [28] found that applying DEET (diethyl-3-methylbenzamide) to ankles and feet resulted in a mean reduction of 69.2% of bites by *An. arabiensis*.

These findings of reduced biting intensity brought about by access denial to preferred bite sites have considerable implications as a supplementary tool for malaria control. This is especially the case in areas where *An. arabiensis* is the dominant vector, as this species is known to often commence feeding early at night [18,33-37] when most people are socializing outdoors [37], and readily feeds outdoors [38,39]. Insecticide-induced adaptive genetic shifts are also resulting in increasing trends of outdoor biting in other vector species [10,12,13], or where insecticide-induced species shifts favour outdoor-feeders [40,41]. Durrheim & Govere [42] published findings indicating that application of DEET to ankles and feet of rural villagers resulted in a strong reduction in malaria cases at village level. This exploitation of the remarkably convergent biting and feeding behaviour of the primary vector species holds potential for large scale application for improved malaria control; there is considerable opportunity presented here for entrepreneurial development of aesthetically appealing yet inexpensive repellent-impregnated anklets or sandals, which can be handed out or inexpensively purchased by rural inhabitants, in the same way as LLINs are supplied/purchased.

### Other biting attributes contributing to increased risk of malaria infection

It is often stated that *An. gambiae* and *An. funestus* are predominantly indoor-feeders [33,38,43-45], while *An. arabiensis* has a less definite preference and feeds both indoors and outdoors [35,38,45]. The results from this study suggest that in the north-central Uganda populations of *An. gambiae* and *An. funestus*, these two species readily feed both indoors and outdoors; although an apparent majority of bites occurred indoors (67% *An. gambiae* and 72% *An. funestus*), this difference is not statistically significant (P = 0.058). Sinka *et al.* [45] collate findings from studies in recent decades, showing that *An. gambiae* in fact bites almost as much outdoors as indoors. Similarly, the oft-repeated statement that *An. funestus* has a preference for indoor-feeding [45] is not absolute, and that a significant percentage of bites occur outdoors [13,14,35,46].

Perhaps more important however, is what time of night these mosquitoes prefer to bite. Rural people living in rural settings in malaria-endemic regions of Africa in most cases eat and socialize outdoors in the evening, retiring indoors to sleep mostly by 21 h00 to 22 h00 [33,35,37,39], and commonly have exposed lower legs unprotected from mosquito bites (Braack, *pers obs*). Studies have shown that *An. arabiensis* in many locations will commence feeding outdoors in very early evening already [18,33,36,37] while *An. gambiae* and *An. funestus* tend to commence feeding later at night [44,45] when most people have turned indoors. Peak feeding periods when biting is most intense varies considerably in *An. arabiensis*: In some countries or geographic sub-regions this species has a biting peak well before midnight [36,37,39], but elsewhere is most intense in the middle hours of the night [34,35] or in the very early hours of the morning near dawn [18,33]. Most studies report *An. gambiae* to have biting peaks somewhere between late at night to the early hours of the morning [33,39,44-48], although it may vary and even peak during the first half of the night in some locations [48]. A similar pattern of biting occurs in *An funestus*, with a biting peak mostly reported between midnight and early morning [33,44-47,49-54]. It would therefore appear that the risk of malaria infection in early evening is primarily from *An. arabiensis* biting at ankles and feet, whereas indoors the risk is from all three species biting later at night and especially early morning and especially at people sleeping at floor level [Braack, *unpublished data*].

The generalized situation outlined above appears to be becoming more fluid as insecticide pressure from IRS and ITN’s is selecting for populations which are increasingly outdoor feeding [10,12,13,40,41] and to a time when people are available outdoors [14,48]. Collectively, these findings should be cause for concern regarding current heavy emphasis on continental-scale malaria control which relies largely on indoor vector control (IRS, ITN’s) with hardly any effort aimed at reducing outdoor biting and outdoor infection risk.

## Conclusions

Malaria remains one of the biggest health challenges in Africa, and the single biggest cause of early childhood mortality. Efforts to address this challenge are focussed largely on vector control. Such vector control relies almost exclusively on indoor strategies such as IRS and ITN’s. Hardly any attention is given to outdoor infection through outdoor biting. Because three of the main malaria vectors all show a strong preference for biting at very low levels close to the ground, and most people in rural settings of Africa socialize in evening with bare or at best only partially covered lower legs, this predisposes them to being bitten by vectors, and also in very early hours of the morning when women typically emerge to tend to fires and other domestic chores, at a time when vector biting intensity is often at its most intense. Additionally, bed nets are often reserved for use by older persons, whereas young children sleep on the floor without bed nets. In such cases the children are doubly at risk, by not having bed nets and sleeping at a level where most of the biting is targeted.

In summary, the ramifications of our findings include that:

- discouraging access to lower limbs (achievable either by plastic bags or repellent-impregnated anklets and/or sandals or wearing trousers, socks and shoes) at seated or standing people in evening can achieve a major reduction in biting intensity by malaria vector mosquitoes.
- people sleeping at floor level bear a disproportionate risk of being bitten at night because this is the preferred height for feeding by the main vector species; many children in Africa appear to sleep on the floor unprotected by bed nets (Braack, *pers. obs*.) and this must surely contribute to the higher incidence of malaria amongst young children.
- opportunity exists for the development of inexpensive repellent-impregnated anklets and/or sandals to discourage vectors feeding at lower legs under outdoor conditions at night. Equally important would be to encourage the use of bed nets for children sleeping at floor level, or providing them with repellent impregnated blankets or sheets.

## Abbreviations

HLC: Human Landing Catches, of mosquitoes arriving to feed at humans
IRS: Indoor Residual Spraying, of household walls with insecticide
ITN’s: Insecticide Treated Nets
